# Power Control for Direct-Driven Permanent Magnet Wind Generator System with Battery Storage

**DOI:** 10.1155/2014/962374

**Published:** 2014-06-19

**Authors:** Chu Xiao Guang, Kong Ying

**Affiliations:** ^1^School of Electrical Information Automation, QuFu Normal University, Rizhao 276826, China; ^2^School of Medical Information and Technology, Jining Medical University, Rizhao 276826, China

## Abstract

The objective of this paper is to construct a wind generator system (WGS) loss model that addresses the loss of the wind turbine and the generator. It aims to optimize the maximum effective output power and turbine speed. Given that the wind generator system has inertia and is nonlinear, the dynamic model of the wind generator system takes the advantage of the duty of the Buck converter and employs feedback linearization to design the optimized turbine speed tracking controller and the load power controller. According to that, this paper proposes a dual-mode dynamic coordination strategy based on the auxiliary load to reduce the influence of mode conversion on the lifetime of the battery. Optimized speed and power rapid tracking as well as the reduction of redundant power during mode conversion have gone through the test based on a 5 kW wind generator system test platform. The generator output power as the capture target has also been proved to be efficient.

## 1. Introduction

With the depletion of resources and environment, new energy has become a focus for global economic growth and sustainable development. Extensive research has been focused to advance the technologies of power generation systems based on various renewable sources, such as solar energy, geothermal, biomass, fuel cell, and industrial waste heat [1–4]. Wind power is gaining momentum because it is pollution-free and abundant and moves to small-scale and large-scale development [5].

Small-scale wind turbine system is flexible and less investment. It is widely used to provide power for areas with loose settlement and abundant wind resources [6, 7]. However, the fluctuation and intermittence of wind frequently result in system power to be imbalanced. Currently, battery is used to address such imbalance. But the lifespan of a battery is 2-3 years, much shorter than a generator or a wind turbine, challenging the cost of wind power generation. Usually the battery is in the state of overcharge and discharge [8–10]. Limited by voltage of battery, wind turbine cannot be employed to the best. Thus, a low power coefficient and the short life of the battery are crucial problems faced with small-scale wind power generators [11–13].

Wind energy capture has always been a hot issue in researches on wind power generation. Two widely accepted methods are tip speed ratio control and power signal feedback control [11, 14–20]. The best tip speed ratio and the maximum power can be concluded from the power curve provided by producers. The controlling target is clear, and the algorithm is easy. But the capture power does not equal effective output power because of losses in mechanical drive and generator, in particular under large load. Loss in generator will make the effective output power derived from the power curve. Another is the hill-climbing searching method based on the generator output power. It can realize the maximum effective output power control, but it cannot achieve the maximum power point tracking speed and the minimum mechanical impact at the same time. A focus on searching speed will unavoidably enhance the mechanical impact. A focus on tracking accuracy may lead to a slow searching speed [19, 21–26]. The abovementioned methods fail to consider the nonlinear, large inertia and fluctuated nature of the wind generator system. Therefore, this paper hopes to optimize the maximum effective output power and turbine speed and further optimize the maximum output power point tracking by constructing a wind generator system loss model.

In fact, wind turbines cannot always be working on the maximum power tracking. Because of the dynamic change of load and battery, the wind generator system must be equipped with maximum power tracking and the accurate power controlling function. To improve the lifetime of the battery, these two functions are corresponding to two operational modes of the maximum power tracking and the accurate power controlling. Given that the inertia of wind turbines results in a slow reaction, this paper adopts the feedback linearization to design the tracking controller in order to enhance the dynamic tracking speed. As mode conversion is frequent and excess power during the conversion will overcharge the battery [27], this paper proposes a dual-mode dynamic coordination strategy based on auxiliary load.

## 2. Principles of WGS with Battery

**Figure 1:**
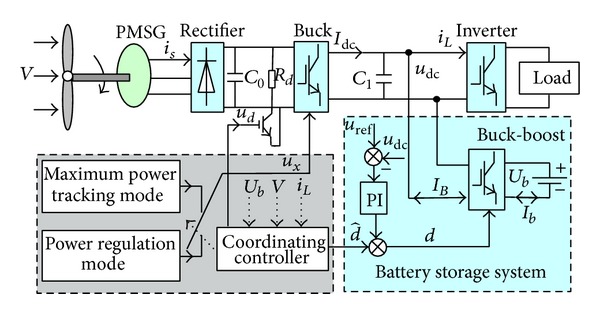
Sketch map of the WGS with battery storage.

The WGS with battery storage is shown in Figure 1, including wind turbine, permanent magnet generator, uncontrolled rectifier, Buck converter, battery storage, and auxiliary load. Buck converter is for adjusting the turbine speed and changing the operation of the wind turbine. Battery storage system manages the charge and discharge by Buck-boost converter. Auxiliary load is mainly for dynamic coordination of mode conversion and for eliminating the excess power during mode conversion.

Because of the dynamic change of the wind speed and the load power, the system is able to operate two operational modes of the maximum power tracking and power controlling. The maximum power tracking is improved on the basis of traditional capture power curve. The tracking strategy is based on effective generator output power. When the output power exceeds the load or the maximum charge power of the battery, the system will switch to the power controlling mode.

The coordinating controller selects the operational mode of the wind generator system according to real-time wind speed, load, and the state of the battery. Given the large inertia and the slow response, this paper proposes a dual-mode dynamic coordination strategy based on auxiliary load to effectively address the imbalanced power during mode conversion.

## 3. Wind Generator System Model

### 3.1. Wind Turbine Model

Small-scale wind turbine usually has fixed pitches and stall-control automatically conducted by blades. It faces the wind by rear control. The capture power of the wind turbine and the torque can be expressed as

(1) Pw=12ρπR2V3,Twind=12ρπR3V2.

In the expression, *ρ* refers to air density, kg/m^3^; *R* is the radius of the blade, m; and *V* refers to wind speed, m/s.

Many kinds of losses in the wind turbine result in a low effective capture power. The maximum coefficient of wind energy is only 0.593. The torque of the loss can be expressed as [13]

(2) $$\begin{array}{c} T_{\text{loss}}=k_{0}V^{2}+k_{1}V\omega +k_{2}\omega^{2}, \end{array}$$

where *ω* refers to turbine speed, rad/sec; *k*_0_~*k*_2_ refer to loss parameters decided by the nature of the turbine.

The effective capture torque is expressed as

(3) $$\begin{array}{c} T_{w}=T_{\text{wind}}-T_{\text{loss}}. \end{array}$$

### 3.2. Permanent Magnet Generator Model

The generator transmits the power through noncontrolled rectifier and Buck converter. And the Buck converter makes it possible to achieve the maximum capture of the effective output power by adjusting the turbine speed.

The electromagnetic torque of the generator and the electromotive force can be expressed as

(4) $$\begin{array}{c} T_{g}=k_{g}i_{s}, \end{array}$$

where *i*_*s*_ is the stator current of the generator, A. Consider

(5) $$\begin{array}{c} E=k_{e}\omega . \end{array}$$

The phase voltage of the generator is expressed as

(6) $$\begin{array}{c} U_{s}=E-L_{s}\frac{di_{s}}{dt}-R_{s}i_{s}. \end{array}$$

In the expression, *k*_*g*_ and *k*_*e*_ are torque coefficient and the electromotive force coefficient, respectively; *L*_*s*_ and *R*_*s*_ are stator inductance and internal resistance.

Because the dynamic response of the turbine is slower than the battery storage system, the voltage *U*_dc_ is considered as constant. *U*_*s*_ is expressed as

(7) $$\begin{array}{c} U_{s}=\frac{\pi U_{\text{dc}}}{3\sqrt{3}}u_{x}. \end{array}$$

If we neglect the loss of converter, the DC output current of the generator is expressed as

(8) $$\begin{array}{c} I_{\text{dc}}=\frac{\pi }{2\sqrt{3}}i_{s}u_{x}. \end{array}$$

### 3.3. Battery Storage System Model

**Figure 2:**
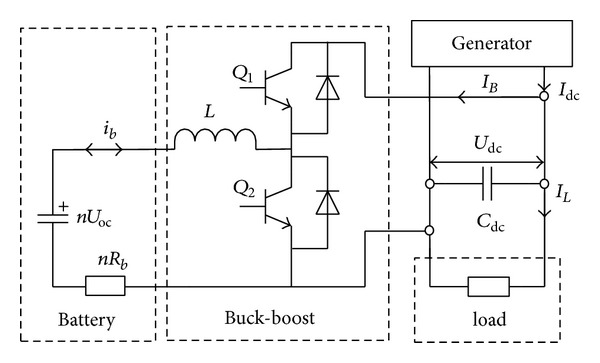
Equivalent circuit of the battery storage system.

The battery storage system is shown in Figure 2. Buck-boost converter is responsible for charge and discharge and stabilizing the DC bus voltage. Then the dynamic function of the DC output current is expressed as

(9) $$\begin{array}{c} C_{\text{dc}}\frac{dU_{\text{dc}}^{}}{dt}=I_{\text{dc}}-I_{B}-I_{L}. \end{array}$$

In the expression, *C*_dc_ refers to the capacitance, *F*, *I*_*L*_ refers to DC load current, and *I*_*B*_ refers to current at the high-voltage side, A.

The battery model is expressed as [28]

(10) $$\begin{array}{c} u_{b}=u_{\text{oc}}-i_{b}R_{b}. \end{array}$$

In the expression, *i*_*b*_, *u*_*b*_, *u*_oc_, and *R*_*b*_ are the battery current, terminal voltage, open circuit voltage, and internal resistance.

If we neglect the power loss of Buck-boost converter, we can get

(11) $$\begin{array}{c} i_{b}=\frac{U_{\text{dc}}I_{B}}{n\left(U_{\text{oc}}-i_{b}R_{b}\right)}. \end{array}$$

To prevent the influence of overcharge and discharge on the lifetime of the battery, the voltage and the current must be limited to

(12) Ub∈[Umin⁡,Umax⁡]ibc,max⁡=0.1C,  ibd,max⁡=0.3C.

In the expression, *U*_max⁡_ and *U*_min⁡_ are the upper limit and lower limit of the voltage. *C* refers to the battery capacity. *i*_bc,max⁡_ and *i*_bd,max⁡_ are the maximum value of charge and discharge, respectively.

Because of the dynamic change of the wind speed and the load, Buck-boost converter can operate two modes, namely, Buck and boost. When *I*_dc_ − *I*_*L*_ ≥ 0 and when *i*_bc_ ≤ *i*_bc,max⁡_ and *U*_*b*_ ≤ *U*_max⁡_, *Q*_2_ is off, and the DC bus voltage is made constant through *Q*_1_ duty, which is the Buck mode; when *I*_dc_ − *I*_*L*_ ≤ 0, *i*_*b*_ ≤ *i*_bd,max⁡_, and *U*_min⁡_ ≤ *U*_*b*_, *Q*_1_ is off, and the DC bus voltage is made constant through *Q*_2_ duty, which is the boost mode.

### 3.4. Wind Generator System Dynamic Model

Suppose the angular speed and the phase current are state variables; we can get the wind generator system dynamic model from expressions (2)~(8), taking the duty *u*_*x*_ of Buck converter as the input. Consider

(13) dωdt=1Jw(12ρπR3V2−k0V2−k1Vω−k2ω2−kgis)disdt=keω−RsisLs−πudc33Lsux.

In the expression, *J*_*w*_ refers to moment of inertia.

## 4. Maximum Effective Generator Power Optimization 

Wind energy capture is decided by power curve provided by producers. But the power curve only indicates the capture power of the turbine rather than effective generator output power. As a matter of fact, losses in turbines do influence the effective capture power and should be considered in the optimized solution to achieve the effective output power and the turbine speed.

The turbine loss power and generator loss power are expressed as

(14) $$\begin{array}{c} P_{w\text{loss}}=T_{\text{loss}}\omega +P_{\text{cu}}+P_{\text{Fe}}. \end{array}$$

In the expression, *P*_cu_ = 3*i*_*s*_^2^ *R*_*s*_; *P*_Fe_ ≈ *E* ^2^/*R*_*f*_ = (*k*_*e*_ *ω*)^2^/*R*_*f*_; *R*_*f*_ is Core loss equivalent resistance.

From expressions (2), (3), (13), and (14), it is easy to get the optimized function for turbine losses:

(15) J=f1ω4+f2ω3+f3ω2+f4ω+f5s.t  0≤ω≤ωmax⁡.

In the expression

(16) f1=−Rsk22kg2f2=−k2−2k1k2RsVkg2f3=−k1V−ke2Rf+(2ak2Rs−k12RsV2−2k0k2RsV2)kg2f4=a−k0V2+(2ak1RsV−2k0k1RsV3)kg2f5=(2ak0RsV2−a2Rs−k0RsV4)kg2a=0.5ρπR3V2.

By *dJ*/*dω* = 0, *ω*_op_ can be acquired by

(17) $$\begin{array}{c} f\left(\omega_{\text{op}}\right)=4f_{1}{\omega_{\text{op}}}^{3}+3f_{2}{\omega_{\text{op}}}^{2}+2f_{3}\omega_{\text{op}}+f_{4}=0. \end{array}$$

By Newton-Raphson method, there is

(18) ωop(n+1)=ωop(n)−f(ωop(n))f′(ωop(n))s.t  f′(ωop(n))=12f1ωop(n)2+6f2ωop(n)  +2f3.

The maximum effective generator output power is

(19) $$\begin{array}{c} P_{g}\left(\omega_{\text{op}}\right)=P_{w}\left(\omega_{\text{op}}\right)-P_{w\text{loss}}\left(\omega_{\text{op}}\right). \end{array}$$

**Figure 3:**
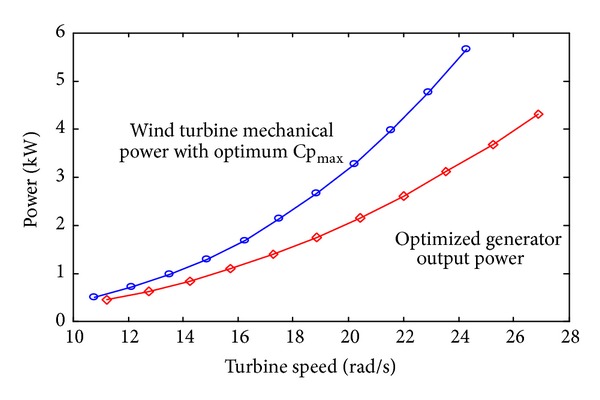
Relation of wind mechanical power and the optimized generator effective power with the turbine speed.

Figure 3 shows the power curve of 5 kW turbines (provided by producers) and the relationship between the generator output power and the optimized turbine speed. As the wind velocity increases, the wind turbine mechanical power (more load) is deviated from the optimized generator output power and both curves have the same trend.

## 5. Dual-Mode Power Control for WGS

**Figure 4:**
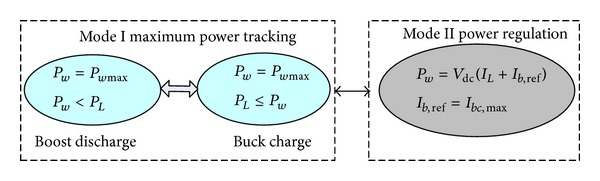
Schematic description of the operational modes.

The maximum effective power tracking mode (Mode I) and power regulation mode (Mode II) are shown in Figure 4. The coordinating controller selects operational modes and completes mode conversion. Given the big inertia of the turbine and the slow response to the mode conversion, in particular when it switches from Mode I to Mode II, there is unavoidably excessive power. Therefore, a dual-mode dynamic coordination strategy based on auxiliary load is proposed to reduce the influence of conversion.

*Mode I*. The battery is in the safety area. The voltage is *U*_*b*_ ∈ [*U*_min⁡_, *U*_max⁡_], and the current is *i*_*b*_ ∈ [*i*_bd,max⁡_, *i*_bc,max⁡_]. The turbine works in the maximum effective output power capture zone. The battery storage system, in accordance with the change of the wind speed and load power, operates at Buck charge or Boost discharge; that is,

(20) Buck  Charge Pw≥PL;  0≤ib≤ibc,max⁡Boost  Discharge Pw≤PL;  ibd,max⁡≤ib<0.

*Mode II*. When the terminal voltage or the charge current exceeds safety limits, that is, when *U*_*b*_ ≥ *U*_max⁡_ or *i*_*b*_ ≥ *i*_bc,max⁡_, the system will switch to the power controlling mode. Under such mode, the turbine will operate for the purpose of reaching the maximum sum of charge power and load power and will reduce the capture power. This is at Buck mode. Suppose the standard power is *P*_*w*_ = *V*_dc_ (*I*_*L*_ + *I*_*b*,ref_). In the expression, *I*_*b*,ref_ refers to the maximum charge current.

### 5.1. Maximum Effective Power Tracking Mode

The wind generator system dynamic model can be converted to [29]

(21) $$\begin{array}{c} \dot{x}=f\left(x\right)+g\left(x\right)u,\;\;y=h\left(x\right). \end{array}$$

In the expression

(22) f(x)=[keω−RsisLs1Jw(0.5ρπR3V2−k0V2−k1Vω−k2ω2−kgis)]g(x)=[−πUdc33Ls0];  h(x)=ω.

Take the optimized turbine speed as output based on the linearization method of input and output to design the controller. Consider

(23) y˙=Lfh(x)+Lgh(x)u=1Jw(0.5ρπR3V2−k0V2−k1Vω−k2ω2−kgis).

As *L*_*g*_ *h*(*x*) = 0, calculate the derivation of $\dot{y}$ and get

(24) $$\begin{array}{c} \ddot{y}=L_{f}\dot{y}+L_{g}\dot{y}u\;=A_{1}\left(t\right)+E_{1}\left(t\right)u_{x}. \end{array}$$

In the expression

(25) A1(t)=−1Jw(kgkeω−kgRsisLs)−1Jw2(keV+2k2ω)×(0.5ρπR3V2−k0V2−kgis−k1Vω−k2ω2)E1(t)=πUdc33JwLs>0.

Let $\ddot{y}=v$; there is the second-order linear system with *v* as the input. The transfer function is *G*(*s*) = 1/*s* ^2^.

**Figure 5:**
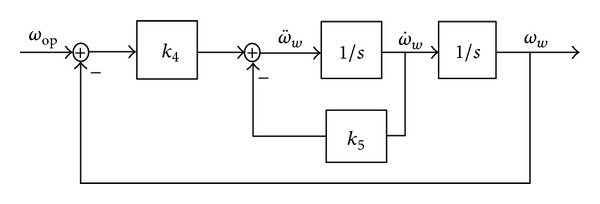
Optimized speed tracking controller.

Given that the system is unstable, the tracking controller with optimized speed based on feedback linearization is shown in Figure 5. Consider

(26) $$\begin{array}{c} v=\ddot{y}=\ddot{\omega }=k_{4}\left(\omega_{\text{ref}}-\omega \right)-k_{5}\dot{\omega }. \end{array}$$

The transfer function of closed-loops system is expressed by

(27) $$\begin{array}{c} G\left(s\right)=\frac{k_{4}}{s^{2}+k_{5}s+k_{4}}. \end{array}$$

According to Routh criterion, the system stability should meet *k*_4_ > 0; *k*_5_ > 0.

To ensure a stable operation and enhance the integral term, for the turbine speed tracking controller, there is

(28) v=k4e−k5e˙+kb∫edt,ux=k4e−k5e˙+kb∫edt−A1(t)E1(t).

In the expression, *e* = *ω* − *ω*_op_.

### 5.2. Power Controlling Mode

When the generator output power exceeds load power, and when the charge current exceeds the safety limits, the system switches from the maximum power tracking mode to power controlling mode and reduces wind energy capture.

Take the generator output power as output and apply the wind generator system mode (21) to feedback linearization. Consider

(29) h(x)=Pg=kgisωw−3is2Rsy˙=∂h∂x[f(x)+g(x)u]=A2(t)+E2(t)u.

In the expression

(30) A2(t)=(kgω−6Rsis)(keω−RsisLs)+kgisJw(0.5ρπR3V2−k0V2−kgis−k1Vω−k2ω2)E2(t)=(kgω−6Rsis)πUdc33Ls>0.

Let $\dot{y}=v_{2}$, here is the first-order linear system with *v*_2_ as the input. It is able to accurately control the turbine power and the power controller is designed as

(31) v2=kge2+kh∫e2dt,ux=kh∫e2dt+kge2−A2(t)E2(t).

In the expression, *k*_*g*_ and *k*_*h*_ are parameters of the controller; *e*_2_ = *P*_*g*_ − *P*_*g*,ref_; and *P*_*g*,ref_ = *i*_*o*,ref_ *U*_dc_ = *i*_bc,max⁡_ *u*_*b*_ + *i*_*L*_ *U*_dc_.

### 5.3. Dynamic Coordination of Mode Conversion

When the wind power exceeds load power and charge power, the system switches from the maximum effective power tracking mode to the power controlling mode. Large inertia of the turbine makes it impossible for the turbine speed to change abruptly. So the excess power is unavoidable during the mode conversion. The dual-mode dynamic coordination strategy realizes the reduction of the charge current.

According to power balance principle, there is

(32) $$\begin{array}{c} P_{g}=P_{g,\text{ref}}+P_{\text{aux}}\\ P_{\text{aux}}=\frac{{\left(U_{\text{dc}}u_{d}\right)}^{2}}{{u_{x}}^{2}R_{d}}=P_{g}-P_{g,\text{ref}}\\ u_{d}=\frac{u_{x}}{U_{\text{dc}}}\sqrt{R_{d}\left(k_{g}i_{s}\omega -3i_{s}^{2}R_{s}-i_{\text{bc},\max}u_{b}-i_{L}U_{\text{dc}}\right)}. \end{array}$$

In the expression, *R*_*d*_ is the resistance of the auxiliary load.

### 5.4. Battery Storage System Controller

The controlling strategy with the combination of DC bus voltage PI control and the duty feed-forward compensation is adopted to effectively control the DC bus voltage.

The charge current is expressed as

(33) $$\begin{array}{c} I_{B}=\frac{\left(I_{o,\max}-I_{L}\right)U_{\text{dc}}}{U_{b}}\;\;I_{o,\max}=\frac{P_{g}\left(\omega_{\text{op}}\right)}{U_{\text{dc}}}. \end{array}$$

From expression (33), there is feed-forward compensation

(34) d^opt=uburef IB>0,  Buckd^opt=uref−uburef IB<0,  Boost.

The DC bus voltage controller, namely, Buck-boost duty (*d*), can be expressed as

(35) $$\begin{array}{c} d=k_{p}\left(u_{\text{ref}}-u\right)+\frac{k_{i}}{s}\left(u_{\text{ref}}-u\right)+{\hat{d}}_{\text{opt}}. \end{array}$$

## 6. Performance Validations

**Figure 6:**
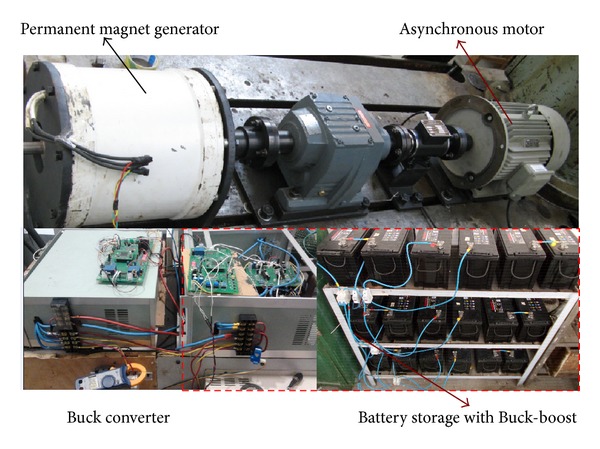
Experiment rig of the wind generator system.

Figure 6 is the experiment rig of 5 kW WGS which is used to evaluate strategy. The wind power simulation system consists of a 7.5 kW Siemens inverter, a 7.5 kW asynchronous motor, a reduction gear, and a 5 kW permanent magnet generator. Battery storage system is formed by Buck-boost and lead acid battery. Parameters are listed in Table 1.

**Figure 7:**
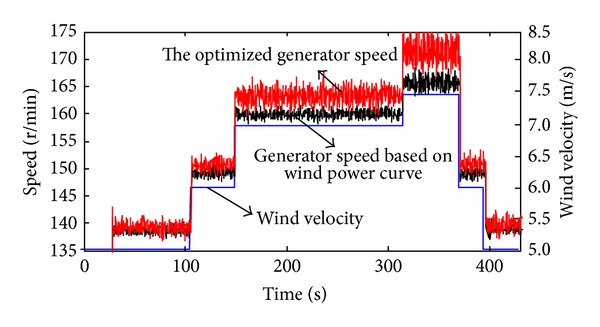
Variation of the wind speed and generator speed in maximum effective power tracking mode.

**Figure 8:**
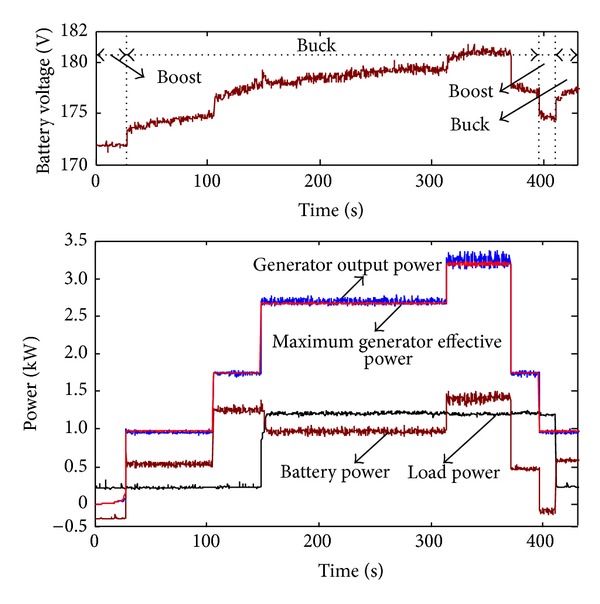
Variation of voltage and power in maximum effective power tracking mode.

**Figure 9:**
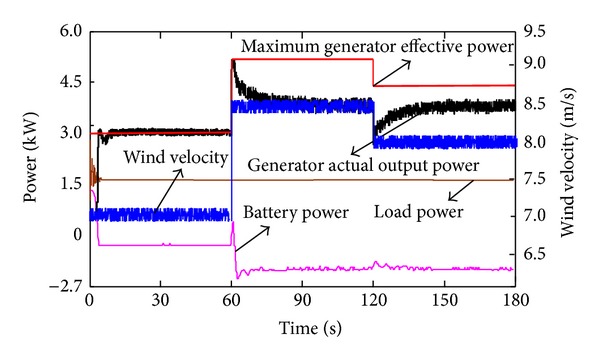
Variation of the wind speed and power in power controlling mode.

Figures 7 and 8 show the operating performance of the wind generator under the maximum effective power tracking mode. The wind speed can randomly change from 5 m/s to 7.5 m/s. The real generator output power in Figure 8 exactly fits the maximum effective output curve. It proves that the tracking and controlling strategy based on optimized turbine speed is effective, and the timely tracking of the maximum effective output can be achieved. Although the 150 s wind speed (from 6 m/s to 7 m/s) and the load (the power increased by 1000 W) both jumped, the system can achieve timely tracking by optimizing the turbine speed to 162 rpm within 1 second, and that the actual output power equals the effective generator output power. The variation of the current in Figure 9 proves that the coordinating controller can rapidly control the battery storage system's charge-discharge mode based on the output current and load current of the generator and can ensure the constancy of the load power.

Before the system startup (within 20 sec), the load power (250 W) is totally provided by battery, and Buck-boost operates in boost mode. The wind turbines start up at 20 s and the wind speed is 5 m/s; generator's output power is 950 W, exceeding the load power of 700 W. Battery storage system operates at the Buck charge mode and rapidly charges the battery by 550 W. The voltage of the battery rises to 175 V within 100 s (Figure 8 shows the battery voltage). At 150 s, the generator's maximum output power reaches 2750 W; the load power increases to 1200 W; and the battery charge power is 850 W. For this purpose, the ascending range of the battery voltage is inferior to the ascending range of voltage when the wind speed was 6 m/s. At 394 s, the wind speed drops to 5 m/s, and the effective output is insufficient to provide the 950 W load power supply; the coordinating controller rapidly changes the battery storage system mode (Boost) and supplies the insufficiency by 250 W power, making the battery voltage 173 V. At 420 s, the load power drops to 250W, and the battery storage system alters the working mode to Buck charge mode; then the battery voltage once more rises to 175 V. As for the abrupt change of the battery voltage during the operation process, it is because the battery voltage is influenced by charge-discharge current.

As shown in Figure 7, the generator maximum output power tracking is achieved by making the generator's optimized speed as the tracking object. Comparing the optimized generator speed and the speed based on power curve, it can be seen that the two vary little at 5 m/s and 6 m/s, but the generator speed increased by 3 rpm and 6 rpm at 7 m/s and 7.5 m/s compared to the speed based on power curve; thereby, it proves the necessity of the wind turbines speed controlling strategy based on generator's loss.

**Figure 10:**
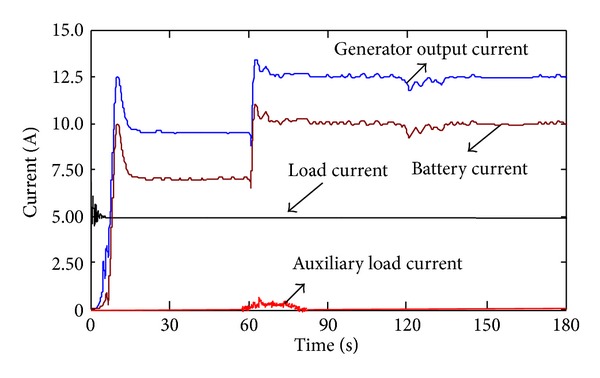
Current variation in power regulation mode.

Figures 9 and 10 show the wind turbines' operation condition when the system is switched from the maximum effective output power tracking mode to the power regulation mode. The system is firstly operated in maximum effective power tracking mode (wind speed 7 m/s), and the charge current of the battery is 7.5 A; the actual output equals the maximum effective output which is 2750 W. At 60 s, the wind speed increases to 8.5 m/s, and the generator's maximum effective output power increases to 5000 W. At this time, in order to prevent the battery from overcharging, the maximum acceptable current and load current are used as criteria and reference to adjust the wind turbines' operating point rapidly, deviating the operating point from the maximum wind-energy capture area. By this, we control the charging current as 10 A as well as guarantee the load power supply. Given that the wind turbine system's great inertia may prevent the mode switch of the dynamic procedure from transient complement, the auxiliary load starts and consumes the surplus power (250 W) by 1 A and finally enables the system to stabilize the charging current as 10 A at around 10 s. At 120 s, the wind speed is reduced to 8 m/s; the generator output power is deviated from the maximum effective output by 500 W and completes the dynamic adjustment within only 15 s as well as maintaining the 10 A charge current. This further proves the effectiveness of power controlling mode.

## 7. Conclusion 

This paper constructs a wind generator system loss model paying attention to wind turbines and generator loss. It aims to optimize the maximum effective output power and turbine speed. Given that the wind generator system has inertia and is nonlinear, this model takes the advantage of the duty of Buck converter and employs feedback linearization to optimize the turbine speed and to design the load power controller. According to that, this paper proposes a dual-mode dynamic coordination strategy based on aiding load.

The test results show that there is a derivation between the turbine speed of the maximum effective output power and that of the traditional power curve. Under huge load, the derivation is as much as 6 rpm. It makes it possible to optimize the turbine speed and effective tracking based on controlling of the maximum charge power and the load power. It addresses the slow reaction to the mode conversion and the overcharge of the battery.

## Figures and Tables

**Table 1:** Wind power generation system parameters.

Parameters of 5 kW WGS	
Generator stator resistance *R*_*s*_/Ω	2.12
Generator stator inductance *L*_*s*_/mH	0.035
Generator torque coefficient *k*_*g*_	18.5
Generator electromotive force coefficient *k*_*e*_	10.59
Generator pole pairs *p*	8
Generator rated power/kW	5
Battery capacity *C*/Ah	100
Battery internal resistance *R*_*b*_/Ω	0.017
Buck-boost power *P*_Boost_/kW	3
Buck converter power *P*_Buck_/kW	5
Turbine loss coefficient *k*_0_	1.3856
Turbine loss coefficient *k*_1_	−0.068
